# A Multi-Ingredient Nutritional Supplement in Combination With Resistance Exercise and High-Intensity Interval Training Improves Cognitive Function and Increases *N*-3 Index in Healthy Older Men: A Randomized Controlled Trial

**DOI:** 10.3389/fnagi.2019.00107

**Published:** 2019-05-09

**Authors:** Kirsten E. Bell, Hanna Fang, Tim Snijders, David J. Allison, Michael A. Zulyniak, Adrian Chabowski, Gianni Parise, Stuart M. Phillips, Jennifer J. Heisz

**Affiliations:** ^1^Department of Kinesiology, University of Waterloo, Waterloo, ON, Canada; ^2^Department of Kinesiology, McMaster University, Hamilton, ON, Canada; ^3^NUTRIM, Department of Human Biology, Maastricht University Medical Centre, Maastricht, Netherlands; ^4^Department of Medicine, McMaster University, Hamilton, ON, Canada; ^5^School of Food Science and Nutrition, University of Leeds, Leeds, United Kingdom; ^6^Department of Physiology, Medical University of Bialystok, Bialystok, Poland

**Keywords:** resistance exercise training, high-intensity interval training, *n*-3 polyunsaturated fatty acids, protein, creatine, vitamin D, calcium

## Abstract

We aimed to evaluate the effect of multi-ingredient nutritional supplementation, with and without exercise training, on cognitive function in healthy older men. Forty-nine sedentary men [age: 73 ± 6 years (mean ± SD); body mass index: 28.5 ± 3.6 kg/m^2^] were randomized to consume a supplement (SUPP *n* = 25; 1500 mg *n*-3 polyunsaturated fatty acids, 30 g whey protein, 2.5 g creatine, 500 IU vitamin D, and 400 mg calcium) or control beverage (CON *n* = 24; 22 g maltodextrin) twice daily for 20 weeks consisting of Phase 1: SUPP/CON followed by Phase 2: 12-week resistance exercise training plus high-intensity interval training, while continuing to consume the study beverages (SUPP/CON + EX). At baseline, 6 weeks, and 19 weeks we assessed cognitive function [Montréal Cognitive Assessment (MOCA)], memory [word recall during the Rey Auditory Verbal Learning Test (RAVLT)], executive functions (working memory inhibition control), and nutrient bioavailability. We did not observe changes to any aspect of cognitive function after Phase 1; however, significant improvements in the following cognitive function outcomes were detected following Phase 2: MOCA scores increased (6 weeks: 23.5 ± 3.3 vs. 19 weeks: 24.4 ± 2.5, *p* = 0.013); number of words recalled during the RAVLT increased (6 weeks: 6.6 ± 3.6 vs. 19 weeks: 7.6 ± 3.8, *p* = 0.047); and reaction time improved (6 weeks: 567 ± 49 ms vs. 19 weeks: 551 ± 51 ms, *p* = 0.002). Although between-group differences in these outcomes were not significant, we observed within-group improvements in composite cognitive function scores over the course of the entire study only in the SUPP group (Δ = 0.58 ± 0.62, *p* = 0.004) but not in the CON group (Δ = 0.31 ± 0.61, *p* = 0.06). We observed a progressive increase in *n*-3 index, and a concomitant decrease in the ratio of arachidonic acid (ARA) to eicosapentaenoic acid (EPA) within erythrocyte plasma membranes, in the SUPP group only. At week 19, *n*-3 index (*r* = 0.49, *p* = 0.02) and the ARA:EPA ratio (*r* = -0.44, *p* = 0.03) were significantly correlated with composite cognitive function scores. Our results show that 12 weeks of RET + HIIT resulted in improved MOCA scores, word recall, and reaction time during an executive functions task; and suggest that a multi-ingredient supplement combined with this exercise training program may improve composite cognitive function scores in older men possibly via supplementation-mediated alterations to *n*-3 PUFA bioavailability.

**Clinical Trial Registration**: http://www.ClinicalTrials.gov, identifier NCT02281331.

## Introduction

Dementia is an incurable and debilitating neurodegenerative disorder that severely impairs cognitive function. Currently, dementia affects approximately 50 million people worldwide, however, this number is expected to reach 75 million by the year 2030 (Prince et al., 2015). The impact of this cognitive impairment is widespread and includes reductions in the ability of affected persons to perform activities of daily living, and increases in the financial and psychological burden on families and caregivers (Alzheimer Society of Canada, 2010). Thus, feasible and effective strategies to promote cognitive function and delay the onset of dementia are needed.

Engaging in regular physical activity is associated with a diminished risk of dementia (Barnes and Yaffe, 2011; Ginis et al., 2017). Shorter term (12–24 weeks) exercise training interventions have been shown to improve aspects of cognitive function in older adults, such as executive functions, memory, and processing speed (Smith et al., 2010). Although most research to date has focused on low-to-moderate intensity aerobic exercise training, higher intensity exercise may yield greater cognitive benefits because it stimulates the production of more factors that promote neuroplasticity, such as BDNF (Schmidt-Kassow et al., 2012; Marquez et al., 2015). HIIT improves memory in young adults (Heisz et al., 2017); however, this modality has yet to be tested in older adults. RET has also been shown to improve cognitive function (Liu-Ambrose and Donaldson, 2009). Importantly, improvements in cognitive function may be enhanced when aerobic and resistance exercise are combined (Colcombe and Kramer, 2003).

Nutrition is another modifiable lifestyle factor associated with brain health in aging (Morris, 2012). Observational studies report that populations consuming Mediterranean-style diets, which contain foods rich in polyunsaturated fatty acids (PUFA) including omega-3 (*n*-3) PUFA, have lower dementia rates (Scarmeas et al., 2006) and better cognitive function (Féart et al., 2009) compared to populations consuming typical Western diets. Furthermore, *n*-3 PUFA supplement may independently prevent cognitive decline in older adults (Karr et al., 2011). Higher intakes of vitamin D (with and without calcium) (Balion et al., 2012), protein (van de Rest et al., 2013), and creatine (Pilatus et al., 2009) are also associated with lower rates of cognitive decline and dementia. However, the effects of *n*-3 PUFA, vitamin D, protein, and creatine supplementation on cognitive function are not consistent across studies, and more research is needed to understand the potential interactive effects of these individual components (Calon, 2011). We propose that combining multiple nutritional supplement with an exercise training program that includes both RET and HIIT components may lead to greater improvements in cognitive function compared to each nutrient or intervention alone (Dziedzic, 2006; Franceschi et al., 2007; Peake et al., 2010).

The objective of this study was to examine the effect of a multi-ingredient nutritional supplement containing *n*-3 PUFA, vitamin D (plus calcium), whey protein, and creatine (Bell et al., 2017b), with and without exercise training, on circulating concentrations of BDNF and cognitive function in a group of healthy older men. A secondary objective was to evaluate whether greater nutrient bioavailability was associated with improvements in cognitive function. We hypothesized that, compared to a control drink, our experimental supplement would independently improve cognitive function as well as enhance the typical beneficial effects of exercise training on cognitive function and BDNF concentrations. We further hypothesized that supplementation would increase vitamin D and *n*-3 PUFA bioavailability; and that individuals with higher circulating *n*-3 PUFA at the end of the intervention would also demonstrate greater improvements in cognitive function.

## Materials and Methods

### Screening and Recruitment

The present study is a secondary analysis from a previously published trial, which examined the effect of multi-nutrient supplementation and exercise training on muscular strength (Bell et al., 2017a,b). Briefly, 49 healthy older men took part in a randomized, double-blind, placebo-controlled parallel group trial that was conducted between December 2014 and September 2016 at McMaster University. Potential participants were eligible for the study if they: were non-smokers ≥ 65 years old; were non-diabetic according to an OGTT, had a BMI in the normal-overweight range, demonstrated normal cardiac function during a maximal exercise stress test; and had not participated in any structured resistance or aerobic exercise training program in the past 6 months. Exclusion criteria included: regular consumption of multi-vitamins, *n*-3 PUFA, whey protein, creatine, calcium, or vitamin D supplements; significant weight loss or gain in the past 6 months; regular use of non-steroidal anti-inflammatory drugs, simvastatin, or anticoagulants; injuries preventing safe participation in an exercise program; diabetes mellitus; cancer; infectious disease; unstable cardiac; and/or gastrointestinal disease.

The primary outcome of the previously published main clinical trial was muscular strength, and as such, sample size was calculated was based on this measure. An increase in leg press isotonic strength of 3.25 kg (standard deviation: 1.5 kg) has been observed during creatine supplementation combined with RET in older adults, versus RET alone (Devries and Phillips, 2014). We assumed a similar response variance in our subjects during a 2-way repeated measures ANOVA; with 80% power and α = 0.05, we estimated needing a minimum of 19 subjects per group. To account for a 20% dropout rate, we aimed to recruit 25 subjects per group (50 subjects total).

**FIGURE 1:**
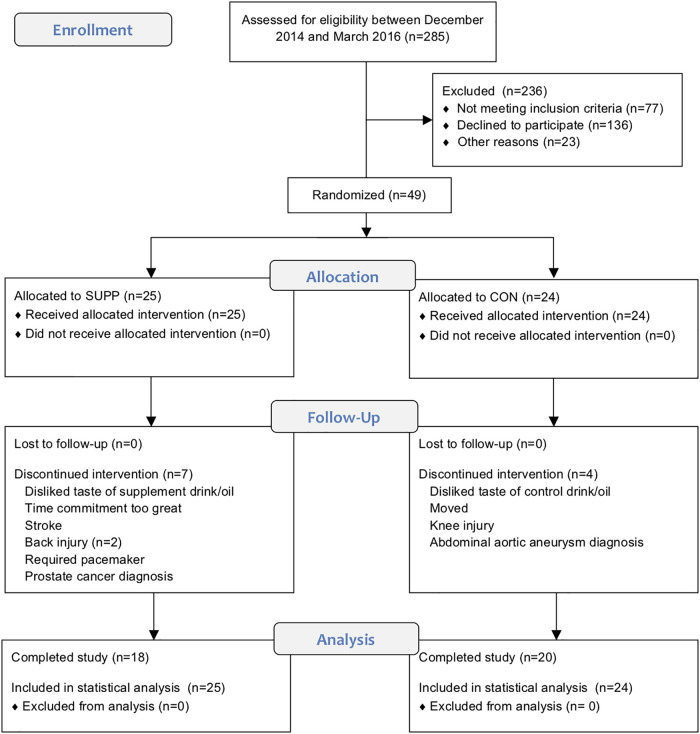
CONSORT flow diagram. This chart depicts the movement of participants through the trial, which we conducted between December 2014 and September 2016.

This study was carried out in accordance with the recommendations of the Canadian Tri-Council policy statement^1^ with written informed consent from all subjects. All subjects gave written informed consent in accordance with the Declaration of Helsinki. The protocol was approved by the Hamilton Integrated Research Ethics Board. This trial was registered at ClinicalTrials.gov (NCT02281331). The CONSORT flow diagram illustrating the movement of subjects through the trial can be found in Figure 1 (see Supplementary File S1 for the accompanying CONSORT checklist).

### Experimental Design

**FIGURE 2:**
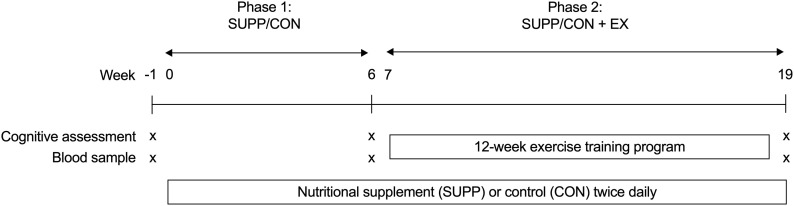
Experimental design. Participants consumed either an experimental supplement (SUPP) or control (CON) beverage twice per day for 20 weeks (weeks 0–19, inclusive). Between weeks 7–18 (inclusive), participants completed a 12-week exercise training program. Exercise training consisted of RET twice weekly (Mondays and Fridays) and HIIT once per week (Wednesdays). At weeks –1 (baseline), 6, and 19 we assessed cognitive function and obtained a blood sample for the measurement of BDNF, 25(OH)D, and erythrocyte plasma membrane phospholipid composition. Phase 1: SUPP/CON took place between weeks 0–6, and Phase 2: SUPP/CON + EX took place between weeks 7–19. SUPP, supplement; CON, control; RET, resistance exercise training; HIIT, high-intensity interval training; BDNF, brain-derived neurotrophic factor; 25(OH)D, 25-hydroxyvitamin D.

Participants were randomly assigned to receive either a multi-ingredient nutritional SUPP (*n* = 25) or a control (CON, *n* = 24) drink for 20 weeks, as depicted in Figure 2. After 6 weeks of consuming their study beverages at home (Phase 1: SUPP/CON), subjects completed a 12-week supervised exercise training program at McMaster University while continuing to consume their assigned beverages (Phase 2: SUPP + EX and CON + EX). We employed a coded (group A versus group B) block randomization scheme (block size: 10 participants) generated using www.randomization.com/ to sequentially allocate subjects to groups in order of enrolment. A key to the randomization code was held by an investigator (SMP) who was not directly involved with subject recruitment, training, or testing. Subjects, as well as investigators who were responsible for recruiting, training, and/or testing subjects, were blind to the individual group assignments. At weeks -1 (baseline), 6, and 19 (post-intervention) we assessed aspects of cognitive function (including verbal memory, executive functions, and processing speed). At each timepoint, we obtained a blood sample the morning after an 8–12 h overnight fast (no food or drink, except for water). Participants refrained from strenuous physical activity for at least 72 h prior to each blood draw. In these blood samples we measured circulating concentrations of BDNF and 25-hydroxyvitamin D [25(OH)D], as well as the phospholipid composition of erythrocyte plasma membranes. Plasma 25(OH)D concentrations and incorporation of eicosapentaenoic acid (EPA) and docosahexaenoic acid (DHA) into erythrocyte plasma membranes reflect vitamin D and *n*-3 PUFA bioavailability, respectively, and indicate compliance with the supplementation protocol as well as overall nutritional health. Our primary outcomes of interest were circulating BDNF concentrations and cognitive function; the secondary outcomes were plasma 25(OH)D concentrations and erythrocyte phospholipid composition.

### Nutritional Supplements

Participants in the SUPP group consumed a multi-ingredient beverage containing: 1,500 mg *n*-3 PUFA (which delivered 700 mg EPA and 445 mg DHA), 30 g whey protein, 500 IU vitamin D, 2.5 g creatine, and 400 mg calcium, twice daily. Participants in the CON group consumed a control beverage containing 22 g of carbohydrate (maltodextrin) twice daily. The exact composition of the SUPP and control drinks has been previously outlined (Bell et al., 2017b). Subjects consumed their first daily beverage within the hour after breakfast, and the second 1 h prior to bed. The control beverages were matched in volume and flavor to the active blend. All study beverages were prepared and labeled in a blinded manner by Infinit Nutrition (Windsor, ON, Canada), and both subjects and researchers were blind to individual group assignments. Participants were instructed not to alter their habitual dietary or physical activity habits (outside of supplementation and exercise sessions included in the protocol) for the duration of the study. To verify adherence to these instructions, participants completed 3-day food records and circulating levels of EPA + DHA and 25(OH)D (see section “Biochemical Analysis”) were evaluated at weeks -1, 6, and 19. At each of these timepoints, participants also wore arm-mounted accelerometers (BodyMedia SenseWear Armband, Cardinal Health Canada; Vaughan, ON, Canada) for 72 h to assess habitual physical activity.

### Exercise Training

From weeks 7 to 18, subjects engaged in a 12-week progressive exercise training program at the Physical Activity Centre of Excellence (PACE) at McMaster University. Details of the exercise training program have been previously published (Bell et al., 2017b). In brief, subjects completed three supervised exercise sessions per week: whole body RET twice per week (Mondays and Fridays) and HIIT on a cycle ergometer once per week (Wednesdays). At every RET session, participants completed two upper body (chest press and horizontal row on Mondays; lateral pulldown and shoulder press on Fridays) and two lower body exercises (leg press and leg extension on both Mondays and Fridays). Training was performed at 80% 1RM (6–8 repetitions) for three sets, with the last set completed until volitional fatigue. We chose this intensity and repetition range because we have previously shown that heavy loads stimulate greater improvements in muscular strength compared to lighter loads in younger men (Mitchell et al., 2012), and the primary objective of the main clinical trial was to induce strength gains. Participants were allowed approximately 2 min of rest between sets.

During their HIIT sessions, subjects completed 10 × 60 s intervals cycling against a workload predetermined to elicit ∼90% maximal heart rate (HRmax) while maintaining a cadence of ≥90 rpm. HRmax was measured during an incremental exercise test on a cycle ergometer at baseline (week -1), as well as re-assessed immediately prior to the initiation of the RET + HIIT program at week 6. We chose this HIIT protocol over the more traditional Wingate-based (i.e., supramaximal) sprint interval training commonly employed in younger adults, because 10 × 60 s HIIT has previously been shown to be well-tolerated by sensitive populations such as sedentary overweight adults (Gillen et al., 2013), and patients with type 2 diabetes (Gillen et al., 2012) and cardiovascular disease (Currie et al., 2013). HR was measured throughout each HIIT session using a chest strap HR monitor (H7 Heart Rate Sensor; Polar Electro Canada; Lachine, QC, Canada). Intervals were interspersed with 60 s of rest where subjects cycled at a self-selected pace against 25 W. All exercise sessions were supervised one-on-one by a member of the research team.

### Cognitive Function Assessments

Cognitive function assessments were conducted one-on-one in a quiet room free from interruptions or distractions, using standardized instructions, and primarily administered by one investigator (HF). Each session began with the MOCA, followed by the RAVLT. The RAVLT requires a 30 min delay between Trials 6 and 7 (the immediate and delayed recall tests, which measure short- and long-term verbal memory, respectively), so during this interval participants completed the executive functions and processing speed assessments on a laptop computer ^2^ (Presentation^®^). The laptop assessments took 15–20 min to complete, and never caused the interval between the RAVLT Trials 6 and 7 to exceed 30 min. All sessions concluded with the RAVLT delayed recall and recognition tests. In total, cognitive assessment sessions lasted approximately 1 h. To minimize the chance of a learning effect at weeks 6 and 19, three versions each of the MOCA and RAVLT were used in a randomized order, so that each participant completed a new version at each visit.

#### Montréal Cognitive Assessment

The MOCA (Nasreddine et al., 2005) evaluates a range of cognitive functions including executive functions, memory, language, and orientation, and may be used to identify signs of MCI. Scores ≥ 26 (out of 30) are considered normal for healthy adults, and scores < 26 indicate potential cognitive impairment.

#### Verbal Memory

During the RAVLT (Rey, 1964) participants were instructed to recall as many words as possible, in any order, from a 15-word test list (List A) after listening to a research assistant read the words aloud. This process was repeated 5 times (Trials 1–5), and each trial was scored based on the number of words correctly recalled. Next, subjects listened to a different 15-word distractor list (List B) and were asked to recall as many words as possible. Directly following this, participants were asked to recall as many words as possible from List A without hearing them again (immediate recall, Trial 6). After a 30 min break (during which participants completed the executive functions and processing speed assessments on a laptop, as described below), participants were once again asked to recall as many words as possible from List A without hearing them again (delayed recall, Trial 7). The participants were then presented with a visual recognition list of 30 words (which included the 15 target words from List A) and asked to circle the target words using a pencil. Memory outcomes analyzed were the sum of words recalled during Trials 1–5 (short-term verbal memory), the number of words remembered during the delayed recall test (Trial 7, long-term verbal memory), as well as the number of words recognized from the visual recognition list.

#### Executive Functions

The Go-NoGo Task assessed working memory inhibition control, a measure of executive functions (Capuana et al., 2012). Participants were presented with 120 uppercase letters in black font appearing one at a time in the center of a white computer screen. They were instructed to press the spacebar (“Go”) when they saw any letter *except* four target letters (“NoGo”: J, D, V, or M). These target letters comprised one third of the total number of trials. A jittered presentation of a blank white screen preceded the appearance of each letter for 500–1,000 ms, and the length of time each letter appeared on screen was 500 ms. Outcomes analyzed were accuracy (i.e., percentage of correct responses) on the “Go” and “No-Go” trials, as well as reaction time on correct “Go” trials.

#### Processing Speed

Processing speed was assessed using a Simple Reaction Time Task (Clark et al., 2015) wherein participants were presented with 60 uppercase letters in black font appearing one at a time in the center of a white computer screen. They were instructed to press the spacebar as quickly as possible anytime a letter appeared on the computer screen. As with the Go-NoGo Task, a jittered presentation of a blank white screen preceded the appearance of each letter for 500–1,000 ms, and the length of time each letter appeared on screen was 500 ms. Accuracy and reaction time were the outcomes analyzed.

### Biochemical Analysis

Whole blood was collected into evacuated tubes coated with lithium heparin, and mixed by inversion. Immediately after collection, tubes were centrifuged and the plasma and erythrocyte layers separated. Fasting plasma 25(OH)D concentrations were measured by radioimmunoassay (DiaSorin Canada Inc.; Mississauga, ON, Canada), and fasting plasma BDNF concentrations were measured using a Quantikine Human Free BDNF ELISA kit (R&D Systems, Inc.; Minneapolis, MN, United States).

An increase in the EPA and DHA content of erythrocyte plasma membranes is detectable within 1 week during supplementation with fish oil-derived *n*-3 PUFA (McGlory et al., 2014). Increased membrane content of EPA, in particular, is associated with decreased ARA, a pro-inflammatory eicosanoid precursor (Calder, 2006). Erythrocyte membrane phospholipid composition was measured from the erythrocyte layer as described previously (Dirks et al., 2016). Briefly, total lipids from the samples were extracted (Folch et al., 1957), and thin layer chromatography was used to separate individual classes of phospholipids. Once isolated, phospholipids were methylated with 1 M methanolic sodium methoxide at room temperature for 10 min (Mahadevappa and Holub, 1987), and the FA composition of each class of phospholipids was analyzed by gas chromatography (Hewlett-Packard 5890 Series II System, equipped with a double flame ionization detector, and Agilent CP-Sil 88 capillary column, 100 m, internal diameter of 0.25 mm) (Nawrocki and Gorski, 2004; Bradley et al., 2008). FAs were identified by comparing retention times to those of a known standard, and absolute amounts of individual FAs were calculated with the aid of an internal standard (pentadecanoic acid), which was added to samples before the methylation process. Total amounts of each phospholipid were determined from the sum of FAs in each fraction. EPA + DHA content was determined by summing the total amount of the EPA and DHA in all phospholipid fractions. *n*-3 PUFA abundance was calculated as the ratio of ARA to EPA (ARA:EPA) and as *n*-3 index as follows:

$$n-3\;index=\left(\frac{EPA+DHA}{total\;FA}\right)\times 100$$

### Calculations

We computed a composite score that included each cognitive outcome positively impacted by the intervention: MOCA scores, delayed recall (Trial 7, long-term verbal memory) performance on the RAVLT, and reaction time for correct “Go” trials during the Go-NoGo Task. Each of these outcomes was first normalized using their respective baseline means and standard deviations. To match the interpretation of other cognitive outcomes (i.e., with higher values indicating better performance), reaction times were reverse-scored. We then averaged together the normalized values for each outcome to create a composite score of composite cognitive function at weeks -1 (baseline), 6, and 19 (post-intervention).

### Statistical Analysis

Statistical analysis was completed using SPSS (IBM SPSS Statistics for Windows, version 23.0; IBM Corp., Armonk, NY, United States). For all measures of cognitive function (MOCA, verbal memory, executive functions, processing speed, and composite cognitive function scores) and all blood analyses [BDNF, plasma 25(OH)D concentrations, and erythrocyte plasma membrane phospholipids] we conducted an intention-to-treat analysis using a linear mixed model with an unstructured covariance matrix, group and time as fixed factors, and subject as a random factor. Age was included as a covariate for all assessments of cognitive function (including composite cognitive function scores) since it is an independent factor related to cognitive performance. In the case of significant group by time interactions, significant between (SUPP or CON) and within (weeks -1, 6, or 19) group differences were identified with Tukey’s *post hoc* test. Based on recommendations for human clinical trials with missing data (Elobeid et al., 2009), all participants (completers as well as participants who withdrew prior to week 6 or week 19 testing) were included in the final analyses, and missing values were not replaced. We examined the effect sizes of the changes in measures of cognitive function using Cohen’s *D*.

As an exploratory sub-analysis, we used one-sample Student’s *t*-tests to evaluate whether the overall change (Δ week 19-baseline) in composite cognitive function scores was significantly different from no change (i.e., zero) in each treatment group (SUPP and CON).

To explore whether consumption of the multi-ingredient experimental supplement was associated with improvements in cognitive function upon completion of the study, we conducted two-tailed Pearson correlations between composite cognitive function scores and the specific nutrients for which bioavailability data was available: *n*-3 PUFA (erythrocyte phospholipid composition) and vitamin D (plasma 25[OH]D_3_ concentrations). In an effort to investigate whether one nutrient may exert a greater effect on cognitive function, we then conducted two-tailed partial correlations: (a) between composite cognitive function scores and erythrocyte phospholipids, while controlling for plasma 25(OH)D; and (b) between composite cognitive function scores and plasma 25(OH)D, while controlling for EPA + DHA content. All correlations were performed using data collected at week 19. Although protein, calcium, and creatine were also included in the experimental supplement, we were unable to assess circulating concentrations of these nutrients. As such, protein, calcium, and creatine were excluded from this exploratory correlation analysis.

Data in text and tables are presented as mean ± SD. For all statistical analyses, significance was accepted as *p* < 0.05.

## Results

### Participants and Compliance

Of the 49 older men randomized, 38 completed the study. Four subjects withdrew prior to week 6 testing (SUPP: *n* = 2; CON: *n* = 2), and seven withdrew partway through the exercise training program, prior to week 19 testing (SUPP: *n* = 5; CON: *n* = 2). Reasons for withdrawal are provided in Figure 1. Participants were 73 ± 6 years (mean ± SD) of age and overweight according to BMI (28.5 ± 3.6 kg/m^2^). There were no significant differences in baseline physical characteristics between the SUPP and CON groups (Table 1). Compliance with the nutrition intervention (assessed via self-report as well as returned sachets) and attendance during the exercise training program were >90% in both the SUPP and CON groups.

**Table 1:** Baseline physical characteristics.

	SUPP (*n* = 25)	CON (*n* = 24)
Age (years)	71 ± 5	74 ± 7
Systolic BP (mmHg)	138 ± 19	138 ± 15
Diastolic BP (mmHg)	78 ± 10	78 ± 8
Body mass (kg)	85.3 ± 12.2	84.5 ± 12.1
Height (m)	1.72 ± 0.07	1.72 ± 0.07
BMI (kg/m^2^)	28.9 ± 3.9	28.1 ± 3.4
Whole body lean mass (kg)	54.0 ± 5.4	54.5 ± 6.7
Whole body fat mass (kg)	28.2 ± 8.6	26.8 ± 6.8
% body fat	33.6 ± 6.4	32.6 ± 4.8
Leg extension 1RM (kg)	27 ± 7	27 ± 7
Leg press 1RM (kg)	77 ± 17	69 ± 21
VO_2_peak (mL/kg/min)	23.8 ± 4.2	24.4 ± 4.6
Peak power (W)	154 ± 25	158 ± 33
Fasting blood glucose (mM)	5.6 ± 0.6	5.8 ± 0.6
2 h blood glucose (mM)	6.8 ± 1.9	7.2 ± 2.1
HOMA-IR	2.1 ± 0.4	2.2 ± 0.5
Total-c (mM)	4.69 ± 1.08	4.83 ± 0.93
LDL-c (mM)	2.74 ± 1.02	2.87 ± 0.86
HDL-c (mM)	1.27 ± 0.31	1.29 ± 0.29
TG (mM)	1.49 ± 0.91	1.50 ± 1.04
Energy intake (kcal/d)	2146 ± 488	2336 ± 553
TEE (kcal/d)	2153 ± 318	2157 ± 627
AEE (kcal/d)	394 ± 258	374 ± 348
Average daily METs	1.3 ± 0.1	1.2 ± 0.3
Depression score^1^	3.2 ± 2.4	3.8 ± 3.6
MOCA score	23.0 ± 2.8	24.2 ± 2.4


*^*1*^Assessed using the Geriatric Depression Scale* (Yesavage et al., 1982). *Data are means ± SD. No significant differences between groups. AEE, active energy expenditure; MET; metabolic equivalent; MOCA, Montréal Cognitive Assessment; SUPP, supplement; CON, control; BP, blood pressure; BMI, body mass index; 1RM, one repetition maximum; VO_*2*_peak, peak oxygen uptake; HOMA-IR; homeostatic model assessment of insulin resistance; c, cholesterol; LDL, low-density lipoprotein; HDL, high-density lipoprotein; TG, triglyceride; TEE, total energy expenditure*.

### Habitual Diet and Physical Activity

Detailed dietary intake data have previously been published (Bell et al., 2017b). Briefly, *n*-3 PUFA, protein, vitamin D, and calcium consumption all increased significantly by week 6 in the SUPP group, and this increase was maintained throughout the rest of the study. Macro- and micronutrient intake was unchanged throughout the study in the CON group. Energy intake increased (*p* = 0.004) and TEE decreased (*p* = 0.006) over time, with no difference between groups. Energy intake increased 10% in both groups at week 6, with no further change following Phase 2. TEE was unchanged in either group at week 6, and decreased 16% following Phase 2.

### Blood Analyses and Nutrient Bioavailability

At baseline, *n*-3 index was significantly lower in the SUPP versus CON group (*p* = 0.038; Table 2), but both groups fell within the range generally associated with moderate cardiometabolic health (i.e., *n*-3 index of 4.1–7.9%) (Harris and Von Schacky, 2004; Block et al., 2008). All other erythrocyte-based outcomes (including EPA + DHA content) were similar between groups at baseline. We observed significant group by time interactions for plasma membrane EPA + DHA content, the ratio of ARA:EPA, and *n*-3 index (all *p* < 0.001). In the SUPP group, EPA + DHA content and *n*-3 index significantly increased 60–70% during Phase 1, and a further 10–20% during Phase 2. The ratio of ARA:EPA was significantly reduced following Phase 1, and this reduction was maintained throughout Phase 2. In the CON group, EPA + DHA content, *n*-3 index, and the ratio of ARA:EPA did not change over the course of the study. At weeks 6 and 19, EPA + DHA content and *n*-3 index were significantly higher, and the ratio of ARA:EPA was significantly lower, in the SUPP vs. CON group. Importantly, in the SUPP group, *n*-3 index was ≥8.0% [the range associated with optimal cardiometabolic health (Harris and Von Schacky, 2004; Block et al., 2008)] at both of these timepoints. Total FA content did not change in either group throughout the study.

**Table 2:** Blood analyses and nutrient bioavailability.

	SUPP			CON		
		
	-1 week	6 weeks	19 weeks	-1 week	6 weeks	19 weeks
Erythrocyte plasma membrane phospholipids						
Total FA (pmol/mg Hb)	10,551 ± 1959	9792 ± 2048	9640 ± 2006	10,098 ± 1810	9259 ± 1790	9523 ± 1684
EPA + DHA (pmol/mg Hb)^2^	496 ± 107^a^	798 ± 294^b*^	882 ± 227^c*^	560 ± 128^a^	471 ± 129^a^	458 ± 123^a^
ARA:EPA^2^	140.4 ± 34.0^a^	68.8 ± 33.6^b*^	55.0 ± 47.9^b*^	127.5 ± 40.9^a^	146.9 ± 42.3^a^	150.6 ± 36.7^a^
*n*-3 index (%)^2^	4.7 ± 0.9^a*^	8.0 ± 1.6^b*^	9.3 ± 2.0^c*^	5.6 ± 1.2^a^	5.1 ± 0.9^a^	4.8 ± 0.9^a^
Plasma 25(OH)D (nM)^2^	44.3 ± 12.8^a^	50.5 ± 14.7^b^	57.1 ± 16.1^c^	37.6 ± 13.8^a^	37.3 ± 12.4^a^	35.6 ± 11.2^a^
BDNF (pg/mL)^1^	391 ± 443^a,b^	384 ± 418^a^	308 ± 240^b^	271 ± 331^a,b^	625 ± 507^a^	273 ± 203^b^


*Data are mean ± SD. Different letters denote significant differences over time within each group. ^∗^Significant difference from CON at that time point. ^*1*^Main effect of time (p = 0.007). ^*2*^Group-by-time interaction (p < 0.001). SUPP, supplement; CON, control; BDNF, brain-derived neurotrophic factor; FA, fatty acid; Hb, hemoglobin; EPA, eicosapentaenoic acid; DHA, docosahexaenoic acid; ARA, arachidonic acid; n-3, omega-3; 25(OH)D, 25-hydroxyvitamin D*.

At baseline, plasma 25(OH)D concentrations were not statistically different between groups (SUPP: 44.3 ± 12.8 nM and CON: 37.6 ± 13.8 nM; Table 2). On average, participants’ vitamin D status was below adequate, but not deficient [adequate: plasma 25(OH)D > 50 nM; deficient: plasma 25(OH)D < 30 nM (Institute of Medicine, 2011)]. We observed a significant group by time interaction (*p* < 0.001) such that in the SUPP group plasma 25(OH)D concentrations increased 14% during Phase 1, and a further 13% during Phase 2. As such, mean vitamin D status in the SUPP group was considered adequate at weeks 6 and 19. In the CON group, plasma 25(OH)D concentrations did not change over the course of the study.

Baseline concentrations of plasma BDNF were not different between groups (Table 2). We observed a main effect of time (*p* = 0.007) whereby BDNF did not change during Phase 1, however, concentrations were reduced approximately 45% following Phase 2, with no difference between groups. Despite this, plasma BDNF was not significantly different between weeks -1 and 19 (*p* = 0.127).

### Cognitive Function

#### Montréal Cognitive Assessment

**FIGURE 3:**
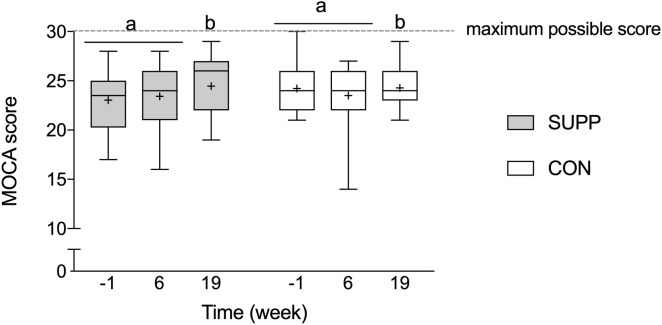
Montréal Cognitive Assessment performance. Scores of 26 or more (out of a possible 30-point total) are considered normal; scores < 26 indicate possible MCI. Boxes (SUPP: gray; CON: white) represent interquartile (25th to 75th percentile) ranges, with the horizontal lines indicating the median. Whiskers represent the maximal and minimal values, and the cross indicates the mean. Dissimilar letters denote changes over time within a given treatment group (SUPP or CON). MOCA, Montréal Cognitive Assessment; MCI, mild cognitive impairment; SUPP, supplement; CON, control.

Prior to beginning the study, mean MOCA scores were below normal cut-points (i.e., <26) in both groups (SUPP: 23.0 ± 2.8 and CON: 24.2 ± 2.4; Figure 3), and there were no between group differences at baseline. We observed a main effect of time whereby no changes occurred over the first 6 weeks of the study (Phase 1), however, following the addition of the 12-week exercise training program (Phase 2) we observed a 4% increase in MOCA scores (SUPP: +7% and CON: +0%; *p* = 0.01). Although we report no significant differences between groups at any point in the study, the effect size of the improvement between weeks 6 and 19 was larger in the SUPP vs. CON group (Cohen’s D: 0.53 vs. 0.02).

#### Verbal Memory

**FIGURE 4:**
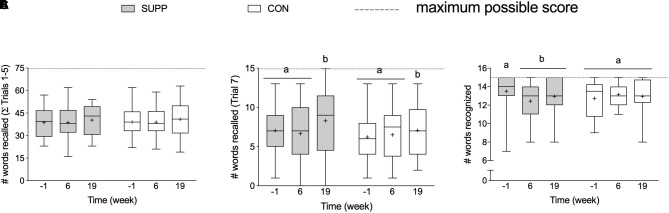
Memory, assessed with the RAVLT. **(A)** Sum of words recalled during Trials 1–5 (out of a possible 75-word total). **(B)** Number of words recalled following a 30 min delay (Trial 7; out of a possible 15-word total). **(C)** Number of words recognized in the 30-word visual recognition list (out of a possible 15-word total). Boxes (SUPP: gray; CON: white) represent interquartile (25th to 75th percentile) ranges, with the horizontal lines indicating the median. Whiskers represent the maximal and minimal values, and the cross indicates the mean. Dissimilar letters denote changes over time within a given treatment group (SUPP or CON). RAVLT, Rey Auditory Verbal Learning Test; SUPP, supplement; CON, control.

At baseline, there were no between group differences in any RAVLT outcomes. We observed a group by time interaction for delayed recognition performance (*p* = 0.02; Figure 4C), where the number of words recognized in the SUPP group decreased significantly by 7% at week 6 with no further change following Phase 2. In the CON group, delayed recognition performance was unchanged throughout the study. We observed a main effect of time for long-term verbal memory: the number of words recalled during Trial 7 (delayed recall; *p* = 0.047) did not change over the first 6 weeks of the study (Phase 1), however, following the addition of the 12-week exercise training program (Phase 2) we observed a 15% increase in the mean number of words recalled (SUPP: +19% and CON: +13%; Figure 4B), with no difference between groups. We did not observe any change in short-term verbal memory; the sum of words recalled during Trials 1–5 did not change in either group over the course of the study (Figure 4A).

#### Executive Functions

**FIGURE 5:**
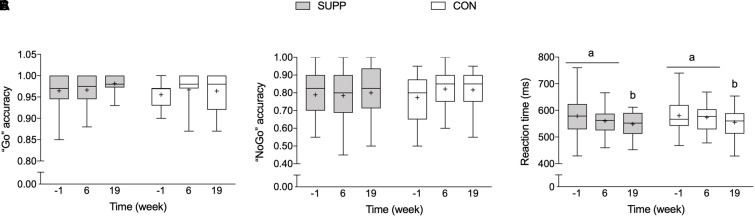
Working memory inhibition control (a measure of executive functions), assessed with the Go-NoGo Task. **(A)** Accuracy (i.e., percentage of correct responses) during “Go” trials. **(B)** Accuracy during “NoGo” trials. **(C)** Reaction time during correct “Go” trials. Boxes (SUPP: gray; CON: white) represent interquartile (25th to 75th percentile) ranges, with the horizontal lines indicating the median. Whiskers represent the maximal and minimal values, and the cross indicates the mean. Dissimilar letters denote changes over time within a given treatment group (SUPP or CON). SUPP, supplement; CON, control.

There were no between group differences in any executive functions outcomes at baseline. We observed a main effect of time for reaction time during correct “Go” trials during the Go-NoGo Task (*p* = 0.002; Figure 5C) whereby no changes occurred over the first 6 weeks of the study (Phase 1), however, following the addition of the 12-week exercise training program (Phase 2) reaction time decreased approximately 5% (SUPP: -5% and CON: -4%). Although we report no significant between group differences following Phase 2, the effect size of the improvement in reaction time during the Go-NoGo Task was larger in the SUPP vs. CON group (Cohen’s *D*: -0.73 vs. -0.44). Accuracy during the “Go” and “NoGo” trials was unchanged throughout the study in either group (Figure 5A,B).

#### Processing Speed

Reaction time (SUPP: 289 ± 51 ms and CON: 300 ± 45 ms) and accuracy (SUPP: 99 ± 1% and CON: 99 ± 2%) during the Simple Reaction Time Task were not different between groups at baseline, and did not change in either group over the course of the study (*p* = 0.42 and *p* = 0.43 for reaction time and accuracy, respectively).

#### Composite Cognitive Function

Composite cognitive function scores were similar between groups at baseline. We observed a main effect of time (*p* < 0.001), such that no change occurred during Phase 1, however, scores increased by 0.46 (SUPP: +0.54 and CON: +0.40; *data not shown*) over Phase 2, with no difference between groups.

**FIGURE 6:**
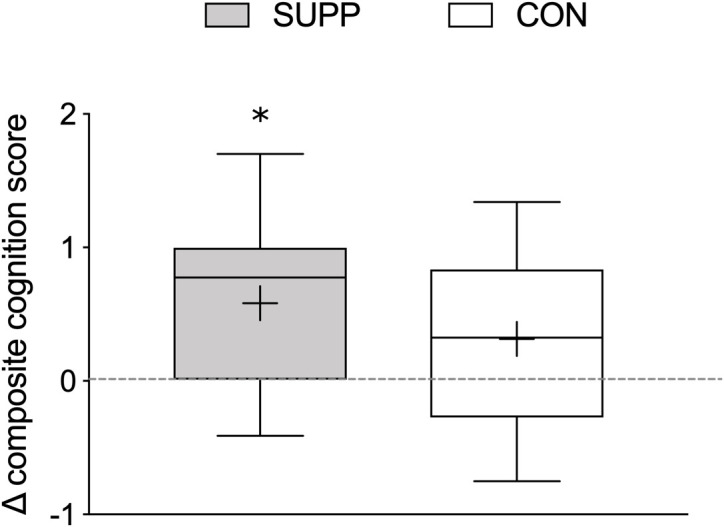
Change in composite cognitive function scores between baseline and 19 weeks. Composite cognitive function scores were calculated using MOCA scores, delayed recall (Trial 7) performance on the RAVLT, and reaction time during correct “Go” trials during the Go-NoGo Task. Boxes (SUPP: gray; CON: white) represent interquartile (25th to 75th percentile) ranges, with the horizontal lines indicating the median. Whiskers represent the maximal and minimal values, and the cross indicates the mean. ^∗^ indicates a significant difference from no change (i.e., zero). SUPP, supplement; CON, control.

In an exploratory sub-analysis we used one-sample *t*-tests to examine whether the change in composite cognitive function scores over the course of the entire study was significantly different from no change (i.e., zero) in each group. We observed a significant increase in composite cognitive function scores in the SUPP group (Δ week 19-baseline: 0.58 ± 0.62; *p* = 0.004; Figure 6); but in the CON group, this increase did not achieve statistical significance (Δ week 19-baseline: 0.31 ± 0.61; *p* = 0.06).

### Correlation Analysis

When subjects were collapsed across treatment group, we observed a trend for a positive correlation between end-of-study composite cognitive function scores and erythrocyte plasma membrane EPA + DHA content, as well as between composite cognitive function scores and *n*-3 index. In line with this, composite cognitive function scores and ARA:EPA at week 19 tended to be negatively correlated (Table 3). There was no relationship between composite cognitive function and circulating 25(OH)D concentrations even after controlling for EPA + DHA content. However, the correlations between composite cognitive function and erythrocyte phospholipid composition were statistically significant after adjusting for plasma 25(OH)D concentrations.

**Table 3:** Pearson and partial correlations between composite cognitive function scores and bioavailable nutrients at week 19 (*n* = 25).

	EPA + DHA content (pmol/mg Hb)	*n*-3 index (%)	ARA:EPA	Plasma 25(OH)D (nM)	Covariate
Composite	0.35 (0.09)	0.39 (0.06)	-0.37 (0.07)	-0.15 (0.43)^a^	*None*
cognitive function	**0.43 (0.04)**	**0.49 (0.02)**	-**0.44 (0.03)**	–	Plasma 25(OH)D
scores	–	0.18 (0.39)	-0.19 (0.38)	-0.31 (0.14)	EPA + DHA content


*^*a*^n = 30. Data are r (p). Significant correlations are bolded.*

## Discussion

We observed that just 12 weeks of multi-modal exercise training improved cognitive function in a group of previously inactive but healthy older men. Bioavailability of certain components of the experimental supplement (*n*-3 PUFA and vitamin D) increased following 6 weeks of supplementation alone and improved further with the addition of exercise training, yet remained unchanged in the control group. Importantly, upon completion of the exercise training program (at week 19), higher bioavailability of *n*-3 PUFA was associated with greater overall cognitive abilities. Taken together, our results show that 12 weeks of RET + HIIT improved MOCA scores, long-term verbal memory, and executive functions in healthy older men; and suggest that multi-ingredient nutritional supplementation may have contributed to superior improvements in cognitive function following exercise training, possibly via an EPA- or DHA-dependent mechanism.

Consistent with prior research, the specific aspects of cognitive function that benefited from our exercise training intervention were executive functions and long-term verbal memory (Smith et al., 2010). Reaction time became faster for the more complex executive functions task (the Go-No-Go Task) but not for the Simple Reaction Time Task (Smiley-Oyen et al., 2008). This may reflect a ceiling effect for the Simple Reaction Time Task, which produced consistently high accuracy scores and fast reaction times even at baseline, leaving little room for an intervention-related improvement. We also report a training-related increase in performance on the MOCA, which evaluates a broader range of cognitive functions and may be used as a tool to screen for signs of MCI. At baseline our participants were below normal cutoffs for the MOCA (<26) indicating possible MCI (Nasreddine et al., 2005). Our intervention resulted in average scores that were closer to normal, and in the experimental group the proportion of subjects with MOCA scores < 26 changed from 20/24 (83%) at baseline to 8/17 (47%) at week 19. Conversely, in the control group, this proportion was 16/23 (70%) at baseline and 13/18 (72%) at week 19. Furthermore, the effect sizes of the improvements in the MOCA and executive functions task were larger in the SUPP group. Contrary to our hypothesis, however, we did not detect significant group by time interactions in any of the individual cognitive functions outcomes, most likely due to a lack of statistical power. As such, we performed an exploratory sub-analysis to further probe the relationship between supplementation, exercise, and cognitive function. When the aspects of cognitive function that were positively impacted by exercise training (MOCA scores, long-term verbal memory, and reaction time during the executive functions task) were combined into a composite score, we observed that the change in cognitive performance over the course of the study was significantly greater than zero in the SUPP group, but not the CON group. This suggests that multi-nutrient supplementation may yet be shown to have a beneficial impact on cognitive function (and perhaps an interactive effect with exercise training), however, more studies are needed. We also observed moderate but significant changes to energy intake and expenditure over the course of the study. Total energy intake increased slightly over Phase 1, likely due to supplementation, which added 100–200 kcal per day. The decrease in TEE over exercise training (Phase 2) was most likely due to participants reducing their habitual physical activity outside of the training sessions, possibly due to muscle soreness and/or fatigue. More intriguing, however, are the improvements in cognitive function that occurred over as few as 12 weeks of exercise training.

The relatively rapid change in cognitive function seen in the present study may be related to the potentially additive effects of exercise training combined with our nutritional supplement containing ingredients known to positively impact cognitive function (Scarmeas et al., 2018). After only 6 weeks of supplementation alone we observed increased bioavailability of certain nutrient components of the experimental supplement (*n*-3 PUFA and vitamin D), with no change in the control group. Although these initial changes were not accompanied by improvements in cognitive function, individuals with an erythrocyte plasma membrane phospholipid containing greater EPA and DHA at the end of the study also demonstrated better cognitive performance, after adjusting for circulating vitamin D. These observations suggest, firstly, that *n*-3 PUFA may play a larger role than vitamin D in effecting cognitive improvements in older adults who are not vitamin D deficient. However, since we were unable to measure the bioavailability of the full complement of nutrients in our multi-ingredient supplement, we do not know if *n*-3 PUFA is primarily responsible for any potential nutrition-mediated improvements in cognitive function. Secondly, our findings suggest that cognitive changes may require a longer exposure to nutritional supplementation or the addition of exercise training to become apparent. Improvements in cognitive function were not detected until the end of the study (i.e., after completion of the 12-week RET + HIIT program). As such, we propose that the initial 6 weeks of the study (Phase 1: SUPP/CON) may not have been enough time to alter brain function. Indeed, older adults may need to begin an exercise training program in a comparative state of optimal nutrition (i.e., be “physiologically primed”) in order for cognitive function to improve over such a short period of time. This interpretation is consistent with animal models that show reduced neuroplasticity with aging (Calabrese et al., 2013), which would slow the rate of potential improvements in brain function and cognitive function in response to the beneficial effects of nutrition and exercise. Consumption of the supplement alone for a period of more than 6 weeks would test this hypothesis, and is an area for future research.

Our study raises important questions about the potential interactive effects of multi-ingredient nutritional supplementation and exercise training on cognitive function in healthy older men, but additional studies are required. Although we speculate that our observed improvements in cognitive function were driven by the SUPP group, our statistical analyses were underpowered to detect between group differences. *Post hoc* sample size calculations revealed that upwards of 150 subjects per group would have been necessary to identify such an effect. Furthermore, the experimental design of this study allows us to account for and identify certain components of the multi-ingredient supplement that appear most or least influential but our certainty is limited by the design of our supplement. Our correlation analysis was exploratory in nature, and the relationship between *n*-3 PUFA, vitamin D, and cognitive function should be further scrutinized in future studies. This study was conducted on healthy older men who had the physical and cognitive capabilities to complete a challenging RET + HIIT program. This challenging exercise intervention would likely require modification before it could be applied in physically and/or mentally impaired older adults. It is also impossible to discount the potential beneficial effects of social interaction (due to study assessments and exercise training sessions) on cognitive function. Our placebo beverage was an effective nutritional control, but subjects in the CON group received the same amount of social interaction from members of the research team as participants in the SUPP group. Future studies should include a no-interaction control group to account for these potential social effects. Lastly, including women in our study would improve the generalizability of the results. We excluded women from the main clinical trial (the primary outcome of which was muscular strength) to improve the homogeneity of our sample. Some prior work suggests the cognitive benefits from exercise may be stronger in women than men (Colcombe and Kramer, 2003), so the observed effects may be heightened in a study that included women.

## Conclusion

We report that an exercise training program that combined RET + HIIT improved MOCA scores, long-term verbal memory, and executive functions in a group of older men in as few as 12 weeks. Importantly, the RET + HIIT program was initiated following 6 weeks of prior multi-nutrient supplementation. Our observation of increased *n*-3 PUFA and vitamin D bioavailability in the SUPP group, coupled with a positive correlation between bioavailable *n*-3 PUFA and overall cognitive function, supports the hypothesis that a multi-ingredient nutritional supplement may enhance cognitive adaptations beyond exercise alone. However, this speculation requires support from additional studies. These results may help to inform the development of a dementia invention strategy that uses physical activity with or without nutritional supplementation to improve cognitive functions for the growing number of older persons.

## Data Availability

All relevant data are within the paper and its Supplementary Material Files.

## Ethics Statement

This study was carried out in accordance with the recommendations of the Canadian Tri-Council Policy statement (http://www.pre.ethics.gc.ca/pdf/eng/tcps2/TCPS_2_ FINAL_Web pdf) with written informed consent from all subjects. All subjects gave written informed consent in accordance with the Declaration of Helsinki. The protocol was approved by the Hamilton Integrated Research Ethics Board.

## Author Contributions

KB, TS, GP, SP, and JH conceived of and designed the study. KB, HF, TS, and MZ collected the data. KB, DJA, AC, and JH analyzed the data. KB and JH wrote the manuscript. All authors read and approved the final version of this manuscript.

## Conflict of Interest Statement

SP is listed as an inventor on patent (Canadian) 3052324 issued to Exerkine, and a patent (US) 16/182891 pending to Exerkine (but reports no financial gains). SP reports personal fees from Enhanced Recovery (donated to charity), equity from Exerkine (all proceeds donated to charity), outside the submitted work. The remaining authors declare that the research was conducted in the absence of any commercial or financial relationships that could be construed as a potential conflict of interest.

## Footnotes

1 http://www.pre.ethics.gc.ca/pdf/eng/tcps2/TCPS_2_FINAL_Web.pdf

2 www.neurobs.com

## Abbreviations

1RM: one repetition maximum
ARA: arachidonic acid
BDNF: brain-derived neurotrophic factor
BMI: body mass index
BP: blood pressure
CON: control
DHA: docosahexaenoic acid
EPA: eicosapentaenoic acid
FA: fatty acid
HDL-c: high-density lipoprotein cholesterol
HIIT: high-intensity interval training
HOMA-IR: homeostatic model of assessment of insulin resistance
IL-6: interleukin-6
LDL-c: low-density lipoprotein cholesterol
MCI: mild cognitive impairment
MOCA: Montréal Cognitive Assessment
*n*-3 PUFA: omega-3 polyunsaturated fatty acid
OGTT: oral glucose tolerance test
RAVLT: Rey Auditory Verbal Learning Test
RET: resistance exercise training
SUPP: supplement
TEE: total energy expenditure
TG: triglycerides
TNF-α: tumor necrosis factor alpha
VO_2_peak: peak oxygen uptake
