# Corrigendum: A Multi-Ingredient Nutritional Supplement in Combination With Resistance Exercise and High-Intensity Interval Training Improves Cognitive Function and Increases *N*-3 Index in Healthy Older Men: A Randomized Controlled Trial

**DOI:** 10.3389/fnagi.2020.611387

**Published:** 2020-10-27

**Authors:** Kirsten E. Bell, Hanna Fang, Tim Snijders, David J. Allison, Michael A. Zulyniak, Adrian Chabowski, Gianni Parise, Stuart M. Phillips, Jennifer J. Heisz

**Affiliations:** ^1^Department of Kinesiology, University of Waterloo, Waterloo, ON, Canada; ^2^Department of Kinesiology, McMaster University, Hamilton, ON, Canada; ^3^NUTRIM, Department of Human Biology, Maastricht University Medical Centre, Maastricht, Netherlands; ^4^Department of Medicine, McMaster University, Hamilton, ON, Canada; ^5^School of Food Science and Nutrition, University of Leeds, Leeds, United Kingdom; ^6^Department of Physiology, Medical University of Bialystok, Bialystok, Poland

**Keywords:** resistance exercise training, high-intensity interval training, *n*-3 polyunsaturated fatty acids, protein, creatine, vitamin D, calcium

In the original article, there was an error in Conflict of Interest Statement. The updated statement appears below.

The authors apologize for this error and state that this does not change the scientific conclusions of the article in any way. The original article has been updated.

## Conflict of Interest Statement

SP is listed as an inventor on patent (Canadian) 3052324 issued to Exerkine, and a patent (US) 16/182891 pending to Exerkine (but reports no financial gains). SP reports personal fees from Enhanced Recovery (donated to charity), equity from Exerkine (all proceeds donated to charity), outside the submitted work.

The remaining authors declare that the research was conducted in the absence of any commercial or financial relationships that could be construed as a potential conflict of interest.

